# Glucocorticoids, the evolution of the stress-response, and the primate predicament

**DOI:** 10.1016/j.ynstr.2021.100320

**Published:** 2021-03-20

**Authors:** Robert M. Sapolsky

**Affiliations:** Departments of Biological Sciences, Neurology, and Neurosurgery, Stanford University, Gilbert Lab MC 5020, Stanford, CA, 94305-5020, USA

**Keywords:** Stress, Primate psychosocial stress, Evolution

## Abstract

The adrenocortical stress-response is extraordinarily conserved across mammals, birds, fish, reptiles, and amphibians, suggesting that it has been present during the hundreds of millions of years of vertebrate existence. Given that antiquity, it is relatively recent that primate social complexity has evolved to the point that, uniquely, life can be dominated by chronic psychosocial stress. This paper first reviews adrenocortical evolution during vertebrate history. This produces a consistent theme of there being an evolutionary tradeoff between the protective effects of glucocorticoids during an ongoing physical stressor, versus the adverse long-term consequences of excessive glucocorticoid secretion; how this tradeoff is resolved depends on particular life history strategies of populations, species and vertebrate taxa. This contrasts with adrenocortical evolution in socially complex primates, who mal-adaptively activate the classic vertebrate stress-response during chronic psychosocial stress. This emphasizes the rather unique and ongoing selective forces sculpting the stress-response in primates, including humans.

## 1 Introduction

In virtually every review of evolutionary psychology or Darwinian medicine, the point is made that for 99% of hominin history, our species has existed in small, nomadic hunter-gatherer bands. This is meant to contrast with the present, whether that means since the invention of agriculture and unequal material culture, since the Industrial Revolution, or since the invention of Facebook or deep-fried double stuf Oreos. The take-home message is always the same – the transition to the particular contemporary lifestyle has been too rapid for hominin evolution to catch up, and pathology arises from that mismatch. The archetypal example of this is the current obesity epidemic: during that 99% of hominin history, food was often sparse and unpredictable, and it was a rarity for hunger to be regularly satiated. As a result, a history with little evolutionary pressure to develop strong physiological signals of satiety must now contend with the novelty of Westernized diets.

The present paper concerns a time span one-to two-orders of magnitude longer than hominin history, in considering the blink-of-an-eye existence of complex primate sociality in the context of hundreds of millions of years of vertebrate evolution. The rationale for this contrast is the remarkable conservation of the endocrine stress-response across vertebrates. The organization of the hypothalamic-pituitary-adrenocortical axis, the reliance upon a handful of types of glucocorticoid molecules (GCs), the rapid increase in GC secretion in the face of challenge are all conserved across mammals, birds, fish, reptiles and amphibians. As one considers the subtleties and complexities of, say, the interactions of socioeconomic status, familial support, social capital and the psychology of attributional style on stress-related health, it is startling to realize that a vast length of time ago, while evading a predator or pursuing a prey, a dinosaur secreted GCs.

Despite this conservatism and antiquity of the adrenocortical stress-response a point must be made, however – no dinosaur ever worried itself sick from perseverating on the absurd idea that an asteroid might strike earth. For 99% of vertebrate history, the stress-response evolved amid selective pressures related to the likes of grassland predator/prey relations, bird migrations or fish schooling. What is one to make of the relatively recent evolution of the stress-response in social primates that can chronically worry? Much as with Oreo cookies in the context of hominin history, chronic psychosocial stress in primates in the context of vertebrate history produces a picture of evolutionary mismatch.

## 2 Obvious caveats and qualifiers that nonetheless bear repeating

-- Since the days of Selye, there is the inevitable lack of clarity as to whether “stress” refers to the external perturbation or the internal response (Levine 2005). Throughout this paper, “stressor” will refer to an external challenge or the anticipation of it (whether warranted or not), while the “stress-response” refers to the internal response to stressors.
-- An increase in GC secretion is not synonymous with stress, as shown by the daily circadian rhythm of GC concentrations. The non-stress roles of GC secretion have been aptly termed “predictive homeostasis” (Romero et al., 2009) and as will be reviewed, their relevance differs dramatically when considering vertebrate evolution versus recent primate evolution.
-- Stressor-induced GC secretion is not synonymous with the entirety of the stress-response, as would be testily emphasized by any autonomic nervous system expert. The heavy emphasis in the stress literature on GCs (and in this review as well), in part reflects the extraordinary range of GC effects on metabolism, immunity, reproductive physiology, brain function and behavior; however, it also reflects the far greater difficulties in studying the secretion of sympathetic catecholamines, especially in naturalistic settings.

## 3 Perhaps less obvious caveats and qualifiers

-- Stressors are less about the absolute intensity of a challenge than about its degree of discrepancy from the norm. For a bird species migrating between the arctic and the tropics, dealing with intensely cold temperatures that, nevertheless, are precisely what would be expected constitutes a normal workday in the arctic for the adrenocortical axis, whereas an unexpectedly chilly day in the tropics can be a life-threatening allostatic challenge (Wingfield, 2013a; b).
-- Different pathologies can arise from abnormalities in basal GC secretion, versus stress-induced secretion, versus secretion during the recovery period after a stressor abates. Not surprisingly then, evolutionary forces have acted independently on these different components of GC secretion (Vitousek et al., 2019).
-- An array of GC actions involve the familiar direct effects on target tissues in response to an ongoing stressor, and these effects have long dominated thinking about stress. However, it must be remembered that GC effects are often, a) indirect, permissively augmenting the direct actions of other signaling systems (for example, the permissive effects of GCs on sympathetic nervous system function); b) preparative, in anticipation of a stressor (for example, prior to a breeding or migratory season); this harks back to preparative homeostasis, and c) suppressive, reversing the effects of early mediators of the stress-response during the recovery period (such as the stimulatory effects of GCs on appetite) (Sapolsky et al., 2000).
-- As appreciated since the golden age of steroid receptor-ology some decades back, variation in levels of receptors for GCs (as well as in levels of receptor cofactors and transport binding globulins), are at least as important as variation in circulating levels of GCs themselves. Framed in an evolutionary context, there can be instances where selective evolutionary pressure on GC receptors has been greater than on GC levels (Hofmeister and Rubenstein 2016).

This review first considers the evolutionary forces that have sculpted the adrenocortical stress response throughout vertebrate history, before then turning to the forces pertinent to the recent emergence of complex primate sociality. One theme will be the remarkable differences in the types of selective forces in these two realms; another will be the need in both domains to question whether individual differences in aspects of the stress-response translate into meaningful differences in Darwinian fitness.

## 4 The evolution of the adrenocortical stress-response in species that don't get all neurotic

For the 99% of vertebrates who have lived without the capacity for significant psychosocial stress, stressors have consisted of the demands of food acquisition, of avoiding being acquired as someone else's food, the travails of the likes of seasonal migrations, or the abiotic challenges of extreme or unpredictable climate. Despite those commonalities, there is no single profile of adrenocortical function that has been selected for across the vertebrate taxa. Instead, variability within both taxa and species reflects differences in life history trade-offs. These are most readily appreciated in balancing between the selective pressures favoring the *protective effects of GCs* during any given stressor and those favoring *protection from GCs* and their cumulative adverse effects over time. A variety of factors come into play in resolving these trade-offs (of note, approximately 80% of the studies to be discussed here have been of birds, about 15% of mammals, and the remainder of reptiles, amphibians or fish [Schoenle et al., 2021]).

### 4.1 How unpredictable or challenging is a particular ecosystem?

A major source of variability is the severity of an ongoing environmental stressor in the context of the long-term costs of chronic GC overexposure. For example, among American redstarts, a migratory bird, elevated basal GC levels predict survival of birds who overwinter in a harsh environment (i.e., scrubs), but not those who overwinter in a more benign one (mangroves) (Angelier et al., 2009). In numerous vertebrate species, higher stress-induced GC secretion is seen in populations living in higher latitudes, and in more adverse ecosystems with lower net primary productivity (Hau et al., 2010; Eikenaar et al., 2012; Jessop et al., 2013).

### 4.2 How many opportunities do individuals have to mate?

Darwinian fitness is, of course, not about survival of the species or of individuals, but is instead measured in the number of copies of genes left by an individual in the next generation (i.e., Reproductive Success, reflecting the combination of individual selection and kin selection**).** The need for trade-offs regarding GC actions are dramatic in the context of reproduction. On one hand, there is the typical stressfulness of reproduction, reflecting its metabolic costs, the demands of courtship displays that are intrinsically competitive and often agonistic, or the necessities of migrations to mating or spawning grounds. On the other are the well-known anti-reproductive effects of the stress-response, particularly via GCs, meaning that from a reproductive perspective, increased GC secretion during a mating period would be at precisely the wrong time. As such, across a range of vertebrates, higher average GC levels (whether basal or stress-induced) during a breeding season predict lower Reproductive Success, while higher levels before a breeding season predict the opposite (Ouyang et al., 2011; Vitousek et al., 2019).

There has thus been selective pressure in some species to spare organisms from GC exposure during reproductive periods, such that stressors that would normally elicit robust GC secretion no longer do so; as an alternative route for accomplishing the same, heavy GC secretion might still occur, but its effects are countered by increased levels of steroid-binding globulins, thereby blunting the GC signal. An even more refined outcome of selection is shown when the anti-reproductive effects of GCs are selectively suppressed. This is seen in a number of bird species and mammals, including primates, where the gonadal axis becomes selectively resistant to the disruptive effects of GCs only during times of reproductive stress (Table 1, from Wingfield and Sapolsky 2003).

**Table 1:** Potential mechanisms underlying resistance of the hypothalamic-pituitary-gonadal axis to stress (from Wingfield and Sapolsky, 2003).

Mechanism	Examples
A: Blockade at the CNS level: stressors are not perceived as stressful	Lekking species, breeding snow bunting
B: Blockade at the level of the HPA: failure to secrete glucocorticosteriods	Many avian species, breeding Lapland Longspurs, redpolls, garter snakes
C: Blockade at the level of the HPG: resistance of the gonadal axis to glucocorticosteriod actions	Male olive baboons, Arctic songbirds
D: Compensatory stimulatory inputs to the gonad axis to counteract inhibitory glucocorticosteriod actions	Male olive baboons, male Arctic ground squirrels, dark-eyed junco
E: Protection from the actions of glucocoticosteriods by, for example, steroid binding proteins	Tree lizards

The resistance of the gonadal axis to the anti-reproductive effects of GCs should be more pertinent to seasonal breeders, relative to species in which mating opportunities are more evenly dispersed throughout the year (Ricklefs and Wikelski 2002). Moreover, such resistance should be more relevant to some species than others. For example, among birds, breeding season GC levels are lower in species with fewer such lifetime opportunities (O'Reilly and Wingfield 2001). Species where there is only a single opportunity to reproduce in a lifetime can show a particularly dramatic sparing of the reproductive system from the suppressive actions of GCs. The classic example of this is Pacific salmon, which famously migrate from the ocean and then arduously swim upstream in fresh-water rivers to where they were born; there they mate, spawn, and then die shortly thereafter (a phenomenon termed “fast phenoptosis”). This process evokes massive GC secretion that is, in fact, the cause of death. By the time of spawning, GCs have caused extensive gastrointestinal and immunological pathologies, and sufficient weight loss such that the gonads constitute 30% of body weight, and adrenalectomy during migration prevents the programmed death (Robertson and Wexler, 1960, 1962; Dickhoff, 1989). Remarkably, the same general life history strategy and GC involvement has evolved independently in some lampreys, eels, and Australian marsupial mice (Woolley, 1966; Bradley et al., 1975, 1980; Larsen, 1985).

The challenges of subtracting out the anti-reproductive effects from the generalized adrenocortical stress-response can be appreciated on the individual level. In a population of petrel birds studied continuously for decades, the resistance of the gonadal axis to the suppressive effects of stress during breeding seasons becomes more pronounced as individuals age (i.e., have decreased future reproductive potential) (Baker and O'Reilly 2000). As another example, among male savanna baboons, the peak period of reproductive potential is quite transient, corresponding to the typically short tenure in the upper echelon of the dominance hierarchy. Among baboons, not only is testosterone secretion in dominant males resistant to the suppressive effects of GCs seen among lesser-ranking individuals, but dominant males actually increase testosterone secretion at such times (Sapolsky, 1991; see also Beehner et al., 2017).

### 4.3 What is lifespan, body size and metabolic rate like in a particular species?

The evolutionary trade-off between ongoing protective effects of stress-induced GC secretion and potential future pathologies of GC excess is strongly influenced by species lifespan. Being relatively long-lived is, in effect, a prerequisite for chronicity of any sort, including chronic GC hypersecretion and its eventual cost. Thus, the longer the future there typically is for a species, the greater the likelihood of paying a price for cumulative GC exposure. Commensurate with that, across bird species, there has been selection for lower basal and stress levels of GCs in species that are longer-lived (Vitousek et al., 2019). Within vertebrate taxa, species with longer lifespans also tend to have larger bodies and lower metabolic rates, and among both birds and mammals, those traits also independently predict lower basal and stress-induced GC concentrations (Bokony et al., 2009; Hau et al., 2010; Haase et al., 2016; Vitousek et al., 2019).

### 4.4 How does early life stress influence life-long physiology?

Considerable amounts of research have focused on uncovering the mechanisms, epigenetic or otherwise, by which early life experience can cause persistent changes in physiology and behavior, changes that can be life-long or even multigenerational. The particular ability of early life stress to persistently alter features of the future stress-response has been framed in at least two contrasting ways (cf Konner, 2011).

*Developmental Constraint* models focus on how such life-long GC overexposure is deleterious, but is an unavoidable price for the protective effects of GCs at the time of a stressor (Monaghan 2008; Douhard et al., 2014). In effect, the eventual pathologies are the lesser of two evils. The ability of chronic inflammation early in life to hasten inflammatory neurodegeneration in the aging brain is a prime example of an outcome of developmental constraint (Bilbo and Schwarz, 2009).

In contrast, *Predictive Adaptive Response* models view early life stressors as useful predictors of the sorts of stressors likely to be common throughout life, and the life-long consequences of early life activation of the stress-response constitutes adaptive preparation for the repetition of the stressors (Gluckman and Hanson, 2006; Lea et al., 2015). A classic example of this would be the Dutch Hunger Winter phenomenon outside the context of a Westernized diet, where prenatal undernutrition produces a life-long “thrifty metabolism” that is particularly good at absorbing and storing calories (Roseboom et al., 2006). In such cases, stress-induced GC secretion is adaptive for both the present and the future.

Given these conflicting models, is early life adrenocortical activation cumulatively damaging or adaptive? Species lifespan turns out to be a significant predictor. The shorter the lifespan of a species, the greater the odds that early life stressors actually resemble the types of stressors coming later in life. This generates the prediction that early life stress-responses are more likely to have evolved to be Predictive Adaptive Responses in shorter-lived species than in those with long lifespans (Snyder-Mackler et al., 2020), and there is support for this idea (Uller et al., 2013; Botero et al., 2015). In contrast, among individuals in long-lived species (in this case, the European white stork, with a life expectancy of >30 years) more dominated by Developmental Constraints features, excessive GC secretion during stress early in life is predictive of shorter life expectancies (Blas et al., 2007; see Lea et al., 2015 for a primate example of early adversity decreasing fitness). As will be seen, the contrasts between Developmental Constraint and Predictive Adaptive Responses models are particularly relevant when considering socially complex primates.

### 4.5 Do less than optimal profiles of adrenocortical function actually decrease fitness?

To reiterate, evolutionary fitness is not about survival or lifespan, but rather about numbers of copies of genes passed down to subsequent generations. As such, individual or populational differences in adrenocortical function that do not translate into differences in fitness are evolutionarily invisible.

Preceding sections noted examples where context-dependent variability in adrenocortical function does indeed translate into differences in Reproductive Success (Vitousek et al., 2019). Nevertheless, it is questionable how often this is the case across vertebrates, and the lack of clarity about this reflects two very different traditions of thinking about stress. The first is probably more familiar to readers of a journal such as this, which is to study the health consequences of stress with medicalized models, typically involving laboratory animals exposed to severe and unethological stressors. This is a view of life as filled with Developmental Constraints whose eventual pathophysiological prices will always come, and is most pertinent to understanding the consequences of psychosocial stress in humans.

The other tradition revolves around animals in natural settings, exposed to naturalistic stressors (as opposed to the increasingly common anthropogenic stressors). As reviewed below, there is ample evidence to suggest that chronic social stress can decrease fitness in other primates; nonetheless, some authors have emphasized how little actual evidence there is for naturalistic stressors lessening fitness in wild populations (Bonier et al., 2009; Boonstra, 2013; Dantzer et al., 2014). This is for a number of reasons:

-- Least interestingly, it is difficult to demonstrate such a relationship, given the challenges of carrying out longitudinal studies of natural populations.
-- A long life filled with chronic and significant stressors is a rarity in natural populations, because of the unlikelihood of surviving them all; there are only so many times an organism can successfully avoid a predator, and only so many times that a predator can fail at killing a prey before starving to death.
-- Most important is the point that a stressor is more about the discrepancy from an expected norm than the absolute value of the norm itself. This is the idea that a frigidly cold day precisely when it is anticipated in the arctic is less stressful for a migratory bird than an unexpectedly cool day in the tropics. In effect, it is less stressful to put on a heavy overcoat that is predictably on the hook by the front door when going outside midwinter, than to search for a sweater in the back of some closet before going outside on an unexpectedly chilly day midsummer. This is the realm of much of GC actions in natural settings being about predictive homeostatic proactivity, rather than post-hoc reactivity. It is not an unexpected stressor for an arctic ground squirrel to find itself having to get by amid arctic weather, or for a desert tortoise to have to endure arid conditions (moreover, in the primate realm, it is not an unexpected stressor for a socially subordinate baboon to be treated in a subordinating manner day in and out). As another example of this point, there are consistent booms and busts in population size among snowshoe hares, driven by cycles of predation risk; despite this, periods of maximal risk do not constitute pathophysiological stressors (Boonstra, 2013; see also Romero et al., 2015). And as an explicit counter to the medicalization of stress, there are species and circumstances where sustained GC hypersecretion is sufficiently adaptive (in both an immediate and preparatory sense) that it actually enhances cumulative fitness (Bonier et al., 2009).

To summarize the evolutionary forces that have acted upon the adrenocortical axis during vertebrate history: a) The tradeoff between the protective effects of GCs in the face of stressors versus the adverse long-term consequences of GC excess varies by species, populations and individuals as a function of predictability of environmental demands, the frequency and nature of mating opportunities, lifespan, body size and metabolism. B) Throughout the life of some species (and particularly during development), there may not actually be a tradeoff involved, in that the protective effects of GCs during a stressor have evolved to also adaptively prepare organisms for future stressors. C) Whether sustained stress has been decreasing fitness for hundreds of millions of years of vertebrate evolution depends, in a sense, on the ethological appropriateness of labeling an event as a stressor; importantly, what might seem self-evidently stressful to us may merely be the predictable and normal exigencies of everyday life in other species.

This summary positions us to now appreciate just how different evolutionary pressures have played out in social primates.

## 5 Evolution and the capacity for experiencing chronic psychosocial stress

Humans share with other primates a number of deeply fundamental similarities, including long lives, living in stable social groups with individuated relationships, where the strength of social bonds can have at least as much impact on fitness as does social status, and where there is the complexity that today's source of social stress may be an individual who must be cooperated with tomorrow (Silk et al., 2003, 2009, 2010, 2020; Seyfarth and Cheney 2012).

Among other things, these shared traits produce a continuum between humans and other primates as to the social circumstances that can alter GC secretion. Some of these similarities can be remarkably subtle. One concerns the strong, stable personality differences observed in other primates. For example, among wild baboons and independent of rank, higher basal GC levels are observed among the males whose ongoing grooming is most likely to be disrupted by the mere non-threatening presence of a higher-ranking male. Moreover, this personality/endocrine profile is stable over years, and is demonstrable on a day in which there has not been an incidence of ongoing grooming being disrupted under these circumstances. Thus, this is the endocrine picture of a constitutional tendency to see social threat that others do not (Sapolsky and Ray, 1989; Ray and Sapolsky, 1992).

As another example, most dominance interactions among male baboons occur with individuals of adjacent ranks (i.e., where a male who ranks, say, fifth in a hierarchy is most likely to be subordinated by #4, and is most likely to subordinate #6); even among baboons, politics is local. Furthermore, such adjacent interactions are typically the most tense and fraught with the potential for aggression and injury, and are most sensitive to male testosterone levels (Bergman et al., 2006). Occasionally there is a reversal of dominance, where #5 unexpectedly dominates #4, or is dominated by #6. Troop-wide rank instability is a substantial stressor for other primates, elevating average GC levels (Sapolsky, 1983), but rank instability among #’s 4–6 is more subtle. What does it mean if #5 has a dominance reversal with #6? That the latter is gaining on the former. What is the meaning of a reversal between #5 and #4? That the former is gaining on the latter. Reflecting that, high rates of dominance reversals with a male one step below in the hierarchy predicts elevated GC levels, whereas high rates with a male one step above does not (and GC measurement are carried out on mornings where no such interactions have occurred). In other words, there are distinct endocrine profiles of the promise of a promotion and the threat of a demotion that persist well past the time of a particular social interaction (Sapolsky, 1992).

Another demonstration of this continuity comes with the power of female sociality and support. An adult male baboon will occasionally transfer into a troop; such males are often initially aggressive and destabilizing, displacing aggression on females after tense dominance interactions with resident males. At such times, females respond to this social stressor with increased intensity of grooming interactions with other females, and those who already have the most stable and focused grooming relationships show the smallest increases in GC levels (Wittig et al., 2008).

As a final example, if wild baboons are injected with beta-carboline antagonists of benzodiazepine receptors, they transiently burst with anxiety-like behaviors -- frenzies of repetitive self-grooming and scratching, teeth grinding, nose wiping, slapping at the ground or purposeless breaking branches or pulling up grass. This disinhibition of anxiety is most pronounced in subordinate animals, who have the most reasons to have elevated endogenous benzodiazepine signaling. However, that is only seen in troops in which subordinates are subject to high rates of displacement aggression by dominant individuals (Sapolsky and Share, 2004).

Thus, stress-related physiology in other primates can reflect personality differences, the social meaning of a behavior independent of its actual performance, the protective effects of social support networks, or cultural differences in different populations. The continuity with our species should be abundantly clear. (In discussing these studies, it is important to note two things. First, these psychoendocrine correlates among wild primates cannot reveal anything about the direction of causality, if any. However, studies of captive primates, in which social settings can be experimentally manipulated, indicate that social circumstance drives the endocrine changes, rather than the reverse [cf Sapolsky, 2005]. Second, this section is written from a primatological perspective; despite that, similar chronic psychosocial stress occurs in other socially complex species, such as elephants or cetaceans.)

These examples underline the different evolutionary pressures on social primates, as compared to the rest of vertebrates. As discussed, for other vertebrates, there is the perpetual tradeoff between the protective effects of GCs in the face of a present threat balanced with whether there is a long-term cost to chronic GC hypersecretion. But the crucial point is that there is no such tradeoff in these primate instances; in the example of tonically elevated basal GC levels in baboons who consistently perceive events to be threatening when others don't, the point is that there is no actual present-tense threat.

The same is observed in humans, in that depression and human anxiety disorders are pathologies of chronic psychosocial stress, built upon perceived lack of control and predictability, lack of coping outlets, and lack of social support. Importantly, both have long been known to be psychopathologies of chronic overactivation of components of the stress-response, with major depression typically associated with elevated basal GC levels or resistance to GC negative feedback (Gillespie and Nemeroff, 2005), and anxiety disorders typically associated with sympathetic hyperarousal (Hoehn-Saric and McLeod, 1988). As with the other primates, there is no trade-off that must be navigated in these circumstances; the sadness caused by tragedy on the other side of the globe, or the anxiety caused by a rent bill coming in a week, does not constitute a present-tense stressor in the vertebrate sense. A flavor of this is found in the approaches of Cognitive Behavioral Therapy (CBT). Prior trauma that has produced depression or anxiety that permeates present life amid no ongoing trauma represents a cognitive distortion. CBT is an attempt to deconstruct that distortion – yes, it is true that you failed dismally, or were unloved and victimized at points in the past … but that is not the present, you have pathologically overgeneralized. It is, in effect, another way of stating that the evolutionary forces that sculpted the vertebrate stress-response are irrelevant in these primate circumstances.

Invoking evolution in the context of primate psychosocial malaise must raise the question of whether these states actually have fitness consequences. As discussed above, the brutality of an arctic winter is merely quotidian if you are an arctic ground squirrel. Does chronic psychosocial stress actually exact a fitness cost in primates? This question has been raised (Beehner and Bergman, 2017), whether framed as the costs of chronic “stress” or chronic GC overexposure, and to date, there is minimal direct evidence that chronic overexposure to GCs decreases longevity or fitness in a wild primate. However, there is abundant indirect support for this conclusion (Snyder-Mackler et al., 2020). The elevated basal levels of GCs caused by psychosocial stress in other primates is associated with elevated sympathetic tone, reproductive impairments, and overexpression of pro-inflammatory genes (Sapolsky 2005; Tung et al., 2012; Snyder-Mackler et al., 2016). Moreover, superb longitudinal studies of female baboons from a number of different field sites show that chronic psychosocial stressors that give rise to chronic GC overexposure (such as social isolation, low-rank or early adversity [e.g., death of a mother]) decrease both longevity and multi-generational fitness (Wasser et al., 1998; Silk et al., 2003, 2009, 2010; Archie et al., 2014; Cheney et al., 2016; Tung et al., 2016; Zipple et al., 2019). Furthermore, among humans, depression and/or anxiety have marked fitness costs, substantially shortening life expectancy, increasing the risk of cardiovascular disease, immunological dysfunction, reproductive disorders, suicide, substance abuse, and metabolic diseases (cf Reiche et al., 2004; Laursen et al., 2016; Walker and Druss, 2016; Halaris, 2017; Joseph and Golden, 2017). By any measure, chronic psychosocial stress exacts a substantial fitness cost in primates.

## 6 Conclusions

A standard trope is that there has been insufficient time for evolution to have caught up with the costs of a Westernized diet. Similarly, there has been insufficient time for evolution to have caught up with primate social complexity and psychosocial stress.

An optimal profile of the adrenocortical stress-response would consist of low basal GC levels, rapid and substantial stress-induced GC secretion but only in the face of “real” vertebrate stressors, and rapid recovery once stressors abate. As such, an easy prediction is that ongoing evolutionary forces are slowly selecting for this sort of profile, selecting against baboons who see threats that other baboons do not and humans crippled by depression or anxiety. Potentially, we may only be a few million years away from primates and, say, marine iguanas having far more different adrenocortical axes than is presently the case.

However, there is a countervailing force that is relevant. It is maladaptive to be incapacitated by constant anxiety about non-existent threats. In contrast, it can be life-saving for say, someone Jewish, Roma or LGBTQ in Berlin in the 1930s to be among the first to perceive substantial threat and leave. Much research and theorizing have focused on context-dependent evolutionary selection, where the same trait can have diametrically opposite fitness consequences in different settings, and on evolutionary bottle-necks, where some unique circumstance removes nearly all of a population, leaving only those with a previously obscure genetic trait. A combination of these evolutionary forces may be occurring now among we primates, producing a scalloped pattern of long periods of gradual selection against those most prone towards psychosocial stress, punctuated with periods of rapid and severe selection where only the canaries in the mine-shafts of psychosocial stress come out the other end. Context-dependent selection and selective bottlenecks mean that primates will be dealing with the evolutionary consequences of malignant sadness and corrosive worry for epochs to come.

## CRediT authorship contribution statement

**Robert M. Sapolsky:** I, as the author, am solely responsible, Conceptualization, Data curation, Formal analysis, Funding acquisition, Investigation, Methodology, Project administration, Resources, Software, Supervision, Validation, Visualization, and all aspects of writing of paper 210308-017180..

## Declaration of competing interest

I have no conflicts of interest.

## Data Availability

No data was used for the research described in the article.
